# Outcomes and toxicities in patients with diffuse-large B cell lymphoma involving the gastrointestinal tract and digestive organs

**DOI:** 10.3389/fonc.2024.1447020

**Published:** 2024-09-11

**Authors:** Gohar S. Manzar, Elaine E. Cha, Kelsey L. Corrigan, Alison K. Yoder, Benjamin R. Schrank, Lewis F. Nasr, Dai Chihara, Luis Malpica Castillo, Ranjit Nair, Preetesh Jain, Sattva S. Neelapu, Maria A. Rodriguez, Paolo Strati, Loretta J. Nastoupil, Jillian R. Gunther, Bouthaina S. Dabaja, Chelsea C. Pinnix, Susan Y. Wu, Penny Q. Fang

**Affiliations:** ^1^ Department of Radiation Oncology, The University of Texas MD Anderson Cancer Center, Houston, TX, United States; ^2^ Department of Lymphoma/Myeloma, The University of Texas MD Anderson Cancer Center, Houston, TX, United States

**Keywords:** diffuse large B-cell lymphoma, DLBCL, gastrointestinal, GI-DLBCL, radiotherapy, radiation therapy

## Abstract

**Background:**

Diffuse large B-cell lymphoma (DLBCL) involving the gastrointestinal (GI) organs is rare, and real-world outcomes after combined modality therapy (CMT) with systemic therapy (ST) and radiotherapy (RT) are not well-characterized, particularly in the contemporary era. We characterized outcomes in a large cohort of GI-DLBCL patients treated with ST alone or CMT.

**Methods:**

Patients with GI-DLBCL treated at a single institution were retrospectively reviewed. Kaplan-Meier and Cox regression models estimated survival. Multivariable analyses were conducted using the Cox proportional hazards model.

**Results:**

Of 204 patients, gastric involvement was most common (63%). Most presented with early-stage disease (61%). All patients received ST and 65 patients (32%) received RT, 88% as part of first-line CMT. Median dose was 36 Gy (IQR 30.6–39.6) in 18 fractions (IQR 17–22). Median follow-up was 46 months. Five-year overall survival (OS) and progression-free survival (PFS) was 88% and 84%, respectively; complete response (CR) rate was 82%. Improved OS associated with low IPI (*p*=0.001), fewer chemotherapy lines (*p*<0.001), early stage (*p*<0.006), and CR (*p*<0.001). Survival did not differ by RT receipt (*p*>0.25). Only early stage and CR correlated with improved OS on multivariable analysis. Stomach-directed RT vs. RT to other sites correlated with improved PFS and OS (*p*<0.04). Patients with early stage DLBCL treated with CMT in the post-rituximab era had equivalent OS vs. ST alone, even with fewer chemotherapy cycles (*p*<0.02; median of 4 with RT vs. 6 cycles without). Fifty patients had bulky disease (≥7.5 cm), of whom 18 (36%) had early stage disease. Among patients with bulky disease, 5 (10%) developed relapse at the initial site of disease bulk. Four of the 5 patients did not receive consolidative radiation. Among these 4 patients, 3 relapsed only in their initial site of bulky disease. Of 191 patients with luminal GI-DLBCL, *n*=4 (2.1%) developed perforation; only one received RT. Acute Grade 3 toxicities were reported in 41.2% of patients, and 12 (5.8%) patients had late Grade 3 toxicities, 99% attributed to chemotherapy.

**Conclusion:**

GI-DLBCL patients have favorable outcomes after CMT with minimal late toxicity. CMT may be offered with abridged systemic regimens with equivalent outcomes. Stomach directed-RT may mitigate relapse risk associated with incomplete disease response or bulky disease.

## 1 Introduction

Non-Hodgkin lymphoma (NHL) accounts for the majority of hematologic malignancies worldwide, but primary lymphomas originating in the gastrointestinal (GI) tract and digestive organs remain relatively uncommon, constituting 10–15% of NHLs (1, 2). Among primary GI lymphomas, the predominant histologic subtypes are diffuse large B-cell lymphoma (DLBCL) and mucosa-associated lymphoid tissue (MALT) lymphoma.

GI-DLBCL is a unique disease subsite characterized by an often intricate clinical presentation and disease symptoms and risks, variable pathogenesis, complex treatment landscape, with unclear long-term outcomes. Over the past few decades, advances in diagnostic techniques, molecular profiling, and therapeutic strategies have shed light on GI-DLBCL disease heterogeneity and revealed potential avenues for targeted interventions. Treatment approaches for GI-DLBCL have evolved over time. However, due to the heterogeneity of the disease and its varied clinical behavior, optimal treatment strategies remain an area of ongoing research.

Radiation therapy (RT) in appropriately selected patients with GI-DLBCL, can be offered as a part of abridged regimens in patients with early stage disease (3) or to mitigate relapse risk associated with incomplete disease response, high risk, or bulky sites of disease (4–6). While combination immunochemotherapy with rituximab and CHOP (cyclophosphamide, doxorubicin, oncovin, and prednisone) has been the standard treatment for decades, contemporary systemic regimens are rapidly evolving incorporating rituximab, polatuzumab, and immunotherapy and cellular therapy options (3). As systemic treatment continues to improve, real-world evaluation of the use and benefit of combined modality therapy (CMT) with RT is needed. Herein, we aimed to characterize outcomes in patients with GI-DLBCL treated with systemic therapy, with or without RT, in a diverse, large cohort of patients.

## 2 Materials and methods

### 2.1 Study population

We identified patients age ≥18 with DLBCL with primary involvement of the GI tract (stomach, small intestine, large bowel, rectum, gallbladder, esophagus, liver, or pancreas) diagnosed between 1/1988—12/2022 at a single institution. All of the patients included had significant GI tract involvement with lymphoma at initial diagnosis (as opposed to later involvement after initial diagnosis with disease at other site(s). Patients with only mesenteric adenopathy were not included. The retrospective review of these medical records was approved by the Institutional Review Board.

### 2.2 ​​Treatment

Patients typically underwent multidisciplinary evaluation with a recommendation for systemic therapy with or without consolidative RT (defined as RT received ≤90 days after completion of chemotherapy). RT may have also been recommended for patients who needed salvage for persistent/progressive disease after ST. ST was typically CHOP (cyclophosphamide, doxorubicin, oncovin, and prednisone) or EPOCH (etoposide, cyclophosphamide, doxorubicin, oncovin, and prednisone), with rituximab typically incorporated for patients treated in the mid-2000s. Some patients received other systemic therapies. A summary of the STs these patients received is summarized in **Supplementary Table 1**. Treatment with intensity modulated radiation therapy (IMRT)/volumetric modulated arc therapy (VMAT) or 2D/3D conformal radiation was per physician discretion and all radiation treatment plans were reviewed in quality assurance conference. Techniques, treatment processes, dosimetric, planning, quality assurance, and follow-up considerations are summarized in our prior literature (7).

CT simulation was performed in a site-specific manner, with motion management considerations, including either deep inspiration breath hold technique or 4-dimensional simulation with capture of fields of interest across the complete respiratory cycle for abdominal fields. The gross primary tumor volume (GTV) and clinical tumor volume (CTV) were defined, with an expansion to planning target volume to account for patient set-up (PTV). Patients were evaluated weekly during treatment.

### 2.3 Data collection

The following demographic data, disease features, and treatment characteristics were retrieved: age at diagnosis, method of diagnosis, presenting symptoms, comorbidities, ECOG, sex, Ann Arbor disease stage, International Prognostic Index (IPI) score, bulky disease (defined as ≥7.5 cm in maximum dimension), presence of B symptoms, site of disease, type and number of cycles of chemotherapy, response to chemotherapy, response assessment (endoscopy, CT, or PET), use of radiation, radiation treatment details, clinical/radiographic outcomes, site of failure (in relation to radiation fields), and disease and vital status at last follow-up, with cause of death if applicable. Provider-assessed toxicities from on-treatment weekly management visits were graded according to Common Terminology Criteria for Adverse Events (CTCAE) v5.0.

### 2.4 Study variables

Survival was estimated with the Kaplan-Meier method to generate univariate analyses for gender, disease genetics (double-hit i.e. high-grade B cell lymphoma with *Myc* and *Bcl2* and or *Bcl6* translocations vs. double-expressor vs. none), achievement of CR, GCB status, IPI at diagnosis, receipt of chemotherapy vs. chemoradiation, ECOG performance status, Ann Arbor disease stage, site of disease (stomach vs. all others), bulky vs. non-bulky disease, and treatment in either the pre- vs. post-rituximab eras. Cox Regression analysis was used to analyze survival across age at diagnosis, SUV_max_ at diagnosis, number of systemic therapy lines, and disease bulk in cm. For comparison of survival between the post-rituximab and pre-rituximab eras, only patients treated with CHOP or EPOCH were included. This selection was also implemented for comparison of chemotherapy vs. chemoradiation in the cohort, comparison of radiation receipt, and comparison of the number of chemotherapy cycles. Additional *a priori* stratifications included examining selected smaller cohorts to compare the OS and PFS outcomes of chemotherapy vs. chemoradiation: 1) early stage only, 2) advanced stage only, 3) bulky disease patients only, 4) pre-rituximab only, 5) post-rituximab only, 6) early stage disease in the post-rituximab era, and 7) advanced stage disease in the post-rituximab era. These cohort analyses were not uniformly adjusted for stage, except as noted above, or for age at all. Among all patients treated with chemoradiation, we compared receipt of RT to the stomach vs. all other sites as a factor for OS and PFS. We also compared the number of cycles of systemic therapy received by patients if they received RT or not, if they had early vs. advanced disease, separating the pre- and post-rituximab eras. For multivariable analyses, we employed a backward stepwise regression process using only variables that achieved a significance level of *p*<0.2 in univariate analyses, and successively excluding variables that were insignificant or did not converge on multivariable modeling. Multivariable analyses were conducted using the Cox proportional hazards model.

### 2.5 Statistical analysis

Patient baseline characteristics were examined with descriptive statistics. The Chi-square test was used to compare categorical variables. Overall survival (OS) computation involved determining the duration from diagnosis to death resulting from any cause. Progression-free survival (PFS) was calculated by determining the duration from treatment until objective tumor progression or cancer-related death. Kaplan-Meier curves were used to visualize survival trends, and the evaluation of survival disparities was conducted using a log-rank test. The number of cycles of systemic therapy received by patients were analyzed by Mann-Whitney U tests. In terms of patient tracking, their status was marked as “censored” upon reaching the date of the latest follow-up, encountering mortality, or arriving at 1/1/2023, depending on whichever event came first. Data was analyzed by SPSS (IBM, Armonk, NY).

## 3 Results

### 3.1 Patient and tumor characteristics

Of 289 identified patients, 204 patients met inclusion criteria (**Supplementary Figure 1**). Demographic and clinical data are presented in **Table 1**. The median age was 63 (range 52–73) years. Most patients (79.4%) had an ECOG performance status of 0–1 and the majority was male (62.3%). Most patients presented without symptoms (32.4%), though 30.9% presented with abdominal pain, and less commonly with bleeding (13.2%) or luminal obstruction (7.4%). Among 51 patients with GI comorbidities, 49% had GERD or ulcerative disease (**Supplementary Figure 2**). Initial presenting symptoms at diagnosis are noted according to GI-DLBCL location in the stomach, small bowel, or large bowel subsites in **Supplementary Table 2**. Luminal obstruction at initial disease presentation was more prevalent in patients with GI-DLBCL of the small bowel (*p*<0.001) vs. stomach. Otherwise, all other treatment-related complications or presenting symptoms were not significantly different between the subsites (*p*>0.12).

**Table 1:** Patient demographics and treatment characteristics, grouped by early vs. advanced stage DLBCL as well as treatment type.

		All patients, *n*=204 *n* (%)	Early stage, *n*=125 (61.3%)		Advanced stage, *n*=75 (36.8%)	
			Chemotherapy only, *n*=71 (56.8%) *n* (%)	Chemoradiation, *n*=54 (43.2%) *n* (%)	Chemotherapy only, *n*=66 (88.0%) *n* (%)	Chemoradiation, *n*=9 (12.0%) *n* (%)
**Age (yr)**	Median (IQR)	63 (52-73)	62 (50–71)	66 (57–72)	63 (53–73)	73 (71–80)
**Sex**	Male Female	127 (62.3) 77 (37.7)	42 (59.2) 29 (40.8)	37 (68.5) 17 (31.5)	39 (59.1) 27 (40.9)	7 (77.8) 2 (22.2)
**Low grade transformation**	Yes No	26 (12.7) 178 (87.3)	9 (12.7) 62 (87.3)	8 (14.8) 46 (85.2)	7 (10.6) 59 (89.4)	2 (22.2) 7 (77.8)
**Method of diagnosis**	Biopsy Imaging Both Missing	137 (67.2) 12 (5.9) 49 (24.0) 6 (2.9)	53 (74.6) 5 (7.0) 11 (15.5) 2 (2.8)	47 (87.0) 0 (0.0) 7 (13.0) 0 (0.0)	32 (48.5) 6 (9.1) 26 (39.4) 2 (3.0)	3 (33.3) 1 (11.1) 5 (55.6) 0 (0.0)
**Type of diagnosis**	Primary Recurrence Missing	195 (95.6) 9 (4.4) 1 (0.5)	68 (95.8) 3 (4.2) 0 (0.0)	54 (100.0) 0 (0.0) 0 (0.0)	61 (92.4) 5 (7.6) 0 (0.0)	8 (88.9) 1 (11.1) 0 (0.0)
**Staging**	Early Advanced Missing	125 (61.3) 75 (36.8) 4 (2.0)	—	—	—	—
	1 2 3 4 Missing	94 (46.0) 29 (14.2) 8 (3.9) 64 (31.4) 9 (4.4)	50 (70.4) 19 (26.8) — — 2 (2.8)	43 (79.6) 10 (28.5) — — 1 (1.9)	— — 8 (12.1) 55 (83.3) 3 (4.5)	— — 0 (0.0) 9 (100.0) 0 (0.0)
	BE	41 (20.1)	12 (16.9)	4 (7.4)	20 (30.3)	5 (55.6)
**Subtype**	GCB Non-GCB Missing	55 (27.0) 25 (12.3) 124 (60.8)	22 (31.0) 8 (11.3) 41 (57.7)	7 (13.0) 4 (7.4) 43 (79.6)	22 (33.3) 12 (18.2) 32 (48.5)	4 (44.4) 1 (11.1) 4 (44.4)
**Genetics**	DE DH TH	22 (10.8) 7 (3.4) 4 (2.0)	6 (8.5) 2 (2.8) 1 (1.4)	2 (3.7) 0 (0.0) 0 (0.0)	12 (18.2) 3 (4.5) 3 (4.5)	2 (22.2) 2 (22.2) 0 (0.0)
**LDH**	Median (IQR) Missing	442 (297–611) 49 (24.0)	439 (247–511) 18 (25.4)	406 (316–519) 8 (14.8)	499 (317–810) 16 (24.2)	747 (522–1727) 3 (33.3)
**ECOG**	0 1 2 3 4 Missing	60 (29.4) 102 (50.0) 14 (6.9) 6 (2.9) 3 (1.5) 19 (9.3)	24 (33.8) 31 (43.7) 6 (8.5) 3 (4.2) 2 (2.8) 5 (7.0)	17 (31.5) 30 (55.6) 3 (5.6) 2 (3.7) 0 (0.0) 2 (3.7)	17 (25.8) 36 (54.5) 5 (7.6) 0 (0.0) 1 (1.5) 7 (10.6)	2 (22.2) 4 (44.4) 0 (0.0) 1 (11.1) 0 (0.0) 2 (22.2)
**IPI**	0 1 2 3 4 5 Missing	28 (13.7) 59 (28.9) 38 (18.6) 29 (14.2) 11 (5.4) 4 (2.0) 35 (17.6)	12 (16.9) 27 (38.0) 16 (22.5) 2 (2.8) 0 (0.0) 0 (0.0) 14 (19.7)	16 (29.6) 27 (50.0) 6 (11.1) 1 (1.9) 0 (0.0) 0 (0.0) 4 (7.4)	0 (0.0) 5 (7.6) 14 (21.2) 25 (37.9) 8 (12.1) 3 (4.5) 11 (16.7)	0 (0.0) 0 (0.0) 2 (22.2) 1 (11.1) 3 (33.3) 1 (11.1) 2 (22.2)
**Severe presenting symptoms**	Obstruction Bleeding GI pain None Multiple Other Missing	15 (7.4) 27 (13.2) 63 (30.9) 66 (32.4) 7 (3.4) 14 (6.9) 12 (5.9)	9 (12.7) 6 (8.5) 23 (32.4) 21 (29.6) 3 (4.2) 5 (7.0) 4 (5.6)	3 (5.6) 11 (20.4) 14 (25.9) 19 (35.2) 2 (3.7) 4 (7.4) 1 (1.9)	3 (4.5) 10 (15.2) 23 (34.8) 20 (30.3) 2 (3.0) 5 (7.6) 3 (4.5)	0 (0.0) 0 (0.0) 2 (22.2) 6 (66.6) 0 (0.0) 0 (0.0) 1 (11.1)
**Disease bulk**	*Size (cm) Bulky (>7.5 cm) Missing	6 (4–10) 50 (24.5) 79 (38.7)	6 (3–8) 13 (18.3) 30 (42.3)	5 (3–7) 5 (9.3) 29 (53.7)	8 (5–10) 27 (40.9) 15 (22.7)	10 (5–11) 4 (44.4) 2 (22.2)
**Max SUV on pre-therapy PET**	Median (IQR) Missing	23 (15–30) 95 (46.7)	26 (15–34) 35 (49.3)	18 (11–24) 31 (57.4)	24 (16–29) 22 (33.3)	25 (15–28) 3 (33.3)

*Median (IQR).

**Figure 1:**
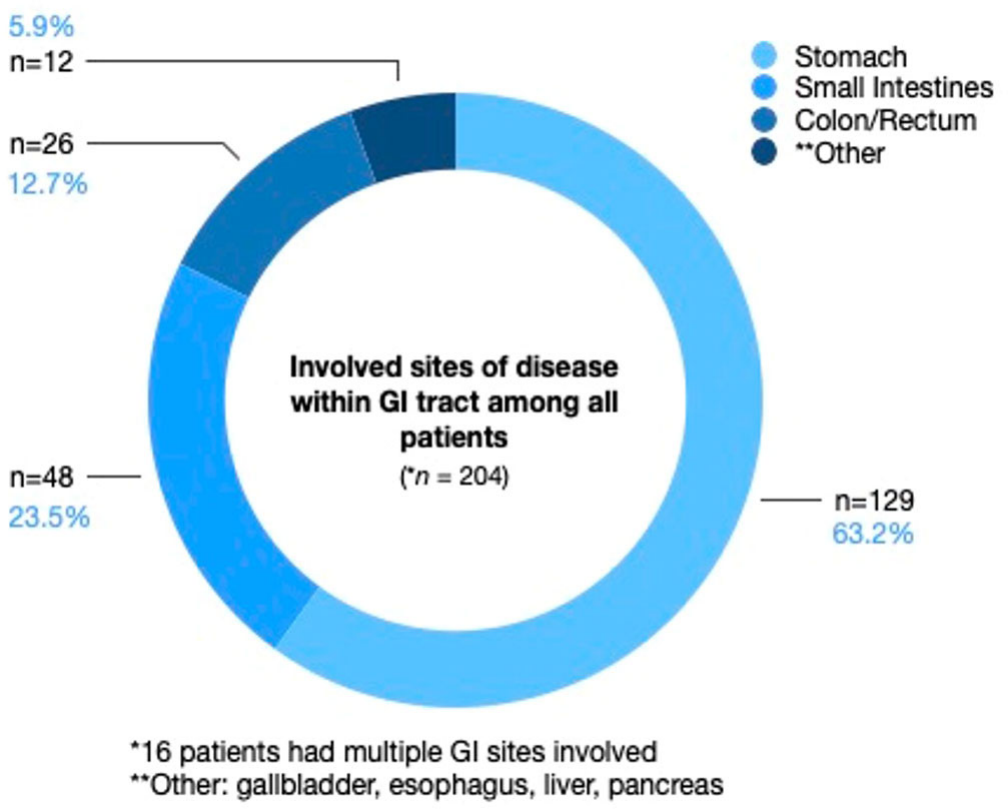
Involved sites of disease within the GI tract among all patients. Stomach was the predominant site of disease, followed by small bowel, and then colon/rectum, followed by other minor sites, including gallbladder, esophagus, liver, and pancreas.

Only 12.7% of patients had transformation to DLBCL from low-grade lymphomas. Most patients had early stage I-II disease (61.3%). Approximately half of the patients (42.6%) had an IPI score of 0–1. Bulky disease (≥7.5 cm) was present in 24.5% of the cohort, more commonly with advanced stage disease (41.3%), compared to 14.4% of patients with early-stage disease. Most patients had disease located in the stomach (63.2%), followed by small bowel (23.5%), colon/rectum (12.7%), and other minor sites, including gallbladder, esophagus, liver, and pancreas, **Figure 1**.

### 3.2 Treatment details

Thirty-eight patients (19%) were treated pre-rituximab; most patients received rituximab (74.0%), **Supplementary Table 1**. CHOP was the most common backbone of treatment (71.1%). Sixty-five patients (31.9%) received RT in this cohort, and of these, the vast majority were patients with early-stage disease (83.1%), **Supplementary Table 3**. Of 66 patients treated with CMT, 33.3% (*n*=22 patients of total *n*=66) were treated in the pre-rituximab era. RT was delivered as part of curative intent upfront treatment for most patients undergoing RT (84.6%). Fifty-one patients (78.5%) received consolidative RT, while 10 (15.4%) had RT targeting residual gross disease and 4 (6.1%) had palliative RT targeting symptomatic extra-abdominal distant sites. The median dose of RT was 36 Gy (IQR: 30.6–39.6) delivered in 18 fractions (IQR: 17–22).

### 3.3 Overview of clinical outcomes for patients with GI-DLBCL

**Figure 2:**
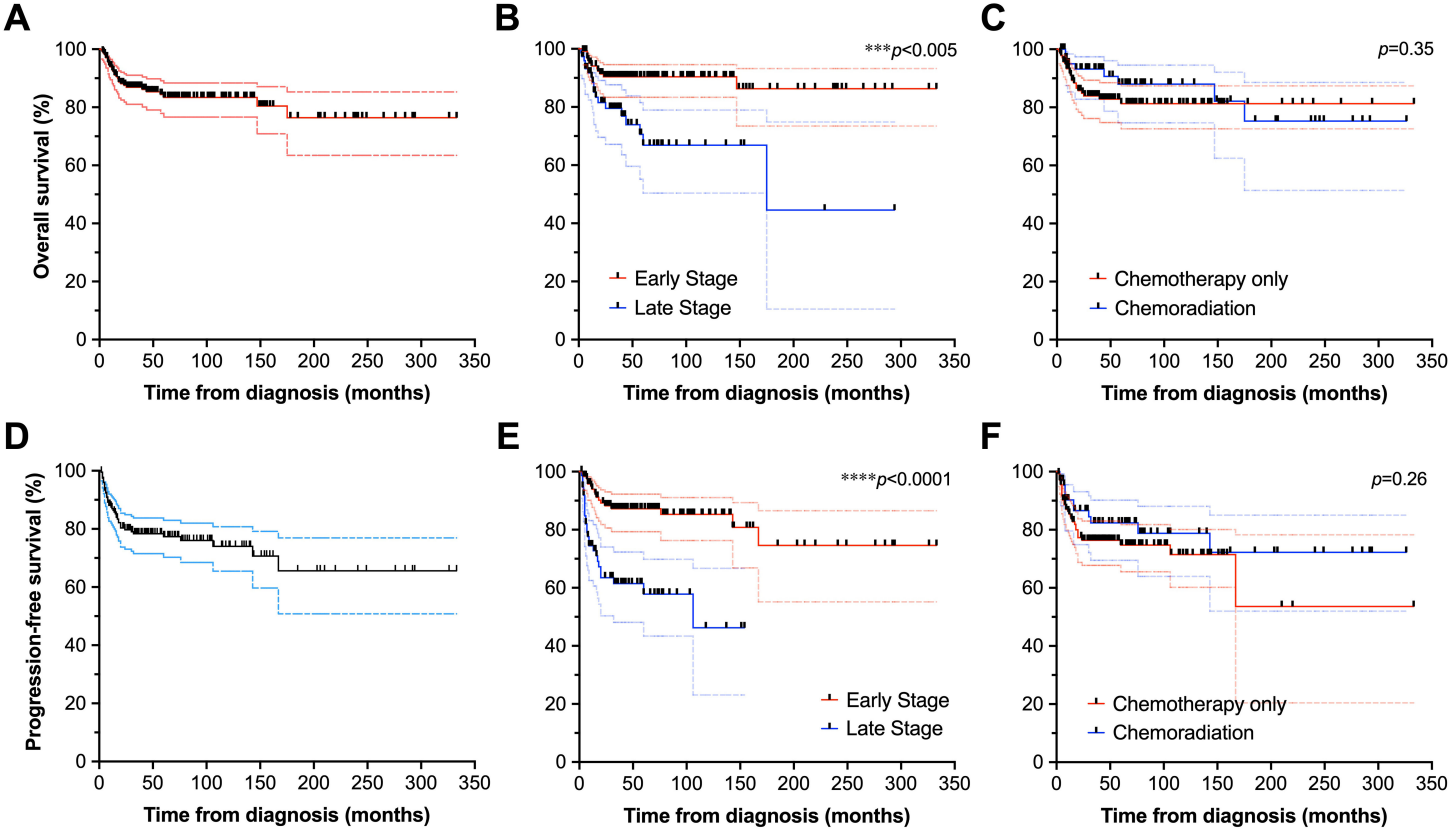
Survival outcomes of patients with DLBCL of the GI tract. Overall survival of the full cohort **(A)**, stratified by stage **(B)**, and receipt of chemotherapy vs. chemoradiation **(C)**. Also depicted are progression-free survival of the full cohort **(D)**, as well as analyses stratified by stage **(E)**, and chemotherapy vs. chemoradiation disposition **(F)**. *p<0.05, **p<0.01, ***p<0.001, ****p<0.0001.

With median follow-up of 46 months (95% confidence interval [CI]: 38–50) from diagnosis, 133 patients (65.2%) were alive at last follow-up, and 71 (34.8%) had expired. Twenty-seven deaths (38.0% of all deaths) were lymphoma-related. The 2-year OS (**Figure 2A**) and PFS (**Figure 2D**) were 96% (91–98) and 91% (86–94); 5-year OS and PFS were 88% (82–92) and 84% (78–88); and 10-year OS and PFS were 84% (78-89) and 77% (70–83), respectively. In palliative cases driven by symptomatology where RT was used (*n*=3), palliation of presenting symptoms was successful in all patients with cessation of bleeding (*n*=1) or resolution of pain (*n*=2). Survival outcomes were excellent for patients treated in the post-rituximab era only (*n*=151), with 2- and 5-year OS (**Supplementary Figure 3A**) of 89% (82-93) and 83% (75-89), respectively, and 2- and 5-year PFS (**Supplementary Figure 3D**) of 79% (71-85) and 76% (68-83), respectively. Among only patients who received CMT (*n*=55), 2-, 5-, and 10-year OS (**Supplementary Figure 4A**) were 95% (83-99), 93% (76-98), and 90% (66-97), and 2-, 5-, and 10-year PFS (**Supplementary Figure 4C**) were 93 (80-97), 89 (74-96), and 82 (59-93), respectively.

### 3.4 Comparison of clinical outcomes by treatment approach for patients with GI-DLBCL

Overall, OS and PFS did not significantly differ between patients who underwent CMT compared to those treated with chemotherapy alone (*p*>0.25 for PFS and OS). Two-year OS (**Figure 2C**) and PFS (**Figure 2F**) were 93% (83–97) and 85% (73–92) for patients undergoing CMT, vs. 93% (83–97) and 87% (80–92) for patients receiving chemotherapy alone. Five-year OS and PFS were 75% (51–89) and 71% (51–84) for patients treated with CMT, and 84% (76–89) and 75% (67–82) for patients treated with chemotherapy alone. Ten-year survival was only evaluable for patients undergoing chemotherapy alone, estimated at 81% (73–87) for OS and 70% (59–79) for PFS.

Among patients treated in the post-rituximab era only, a trend toward improved OS was seen among patients treated with CMT, *p*=0.06, with OS of 97% (78-100) for both 2- and 5-years with CMT and 2- and 5-year OS of 87% (78-92) and 79% (69-98) with systemic therapy alone, respectively, **Supplementary Figure 3C**. Two- and 5-year PFS (**Supplementary Figure 3F**) were 91% (72-97) and 83% (55-94) with CMT and 74% (65-82) and 72% (62-80) with systemic therapy alone, respectively, *p*=0.11.

Disease recurrence occurred in 46 patients (23%). Of these, 60% of recurrences manifested in patients with advanced stage disease at diagnosis. Five of these patients had originally received CMT with RT. Of these 5 CMT patients, 1 patient had in-field recurrence following salvage RT to 20 Gy in 8 fractions. Median time to recurrence was 8.7 months (IQR: 5.2–18.9). Salvage therapy included systemic therapy alone in 50% of patients, 19% CMT using RT, and RT alone for 4.3% of patients.

### 3.5 RT receipt is associated with reduced number of cycles of systemic therapy

**Figure 3:**
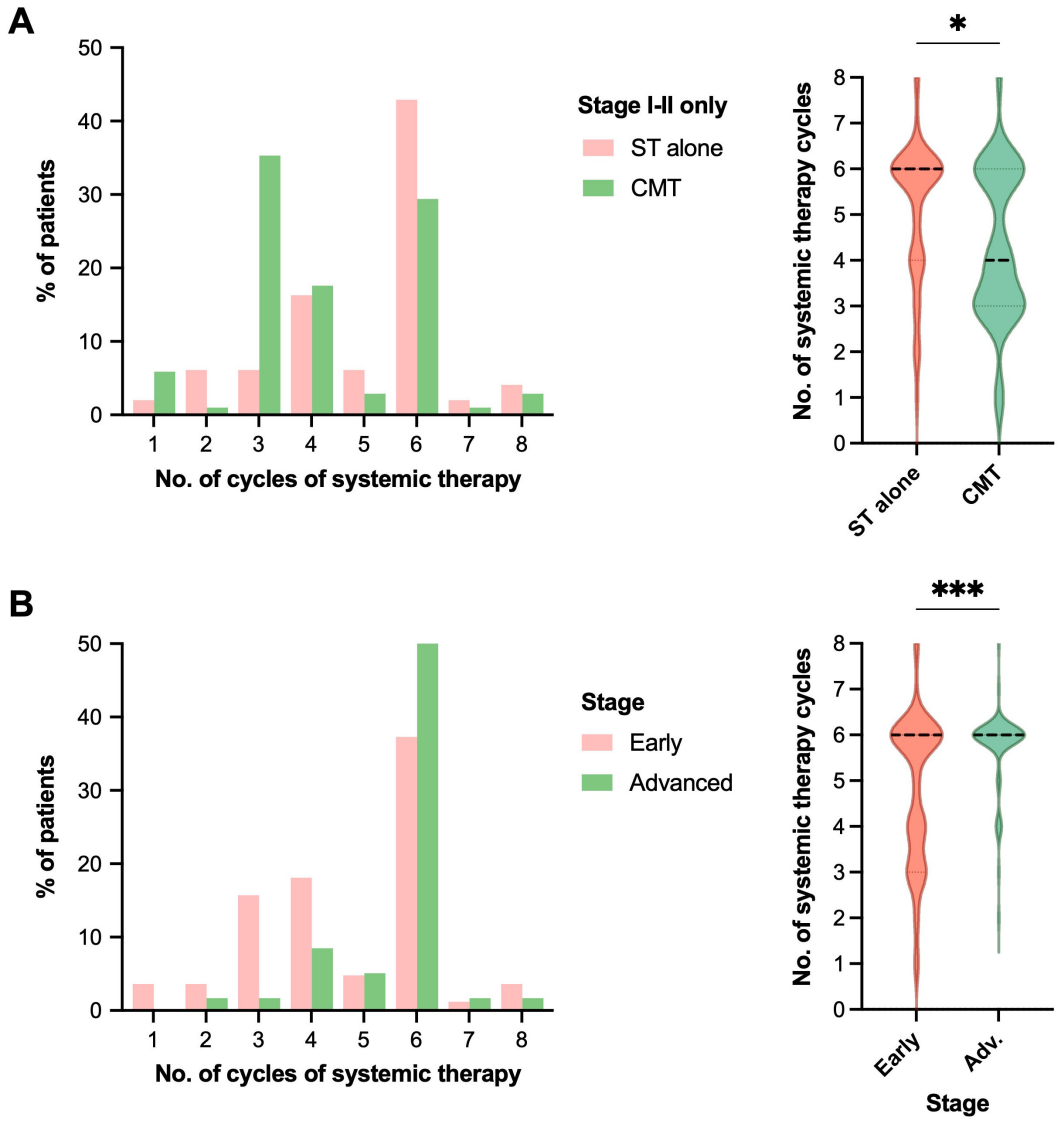
RT receipt and early-stage disease are associated with reduced number of cycles of systemic therapy. Comparison of the number of systemic therapy cycles received by patients in the post-rituximab era in histograms (left) and violin plots (right, for statistical display), comparing **(A)** treatment strategy with either systemic therapy (ST) alone vs. combined modality therapy (CMT) for patients with early-stage disease, or **(B)** limited vs. advanced stage of disease. Statistical analysis was via Mann-Whitney U, *p<0.05, **p<0.01, ***p<0.001. Median represented by the thick dashed line, and quartiles by the thin dashed lines.

Patients treated with CMT for early stage disease received fewer cycles of systemic therapy in the post-rituximab era compared to those treated with systemic therapy only, median (IQR and range) = 4 (3–6 and 1–8) with CMT vs. 6 (4–6 and 1–8) with chemotherapy alone, *p*=0.004 (all stages) and *p*=0.013 (early stage only), **Table 2** and **Figure 3A**. In the post-rituximab era, patients with early stage disease received fewer cycles of chemotherapy compared to patients with advanced stage disease, with a median (IQR and range) number of cycles of 6 (3.5–6 and 1–8) for early stage vs. 6 (6–6 and 2–8) for advanced stage, *p*<0.001, **Table 2** and **Figure 3B**.

**Table 2:** Association of disease stage and treatment category with number of cycles of systemic therapy used for treatment.

Cohort/Factors	*p*-value	No. of cycles of systemic therapy	
**Cohort:** Post-Rituximab **Factor:** Chemo vs. CMT	<0.001	**No RT:** 6 (5–6)	**CMT:** 5 (3–6)
**Cohort:** Post-Rituximab **Factor:** Early vs. Advanced stage	<0.001	**Early:** 6 (3.5–6)	**Advanced:** 6 (no range)
**Cohort:** Pre-Rituximab **Factor:** Chemo vs. CMT	0.352	**No RT:** 6 (no range)	**CMT:** 6 (no range)
**Cohort:** Pre-Rituximab **Factor:** Early vs. Advanced stage	0.534	**Early:** 6 (no range)	**Advanced:** 6 (no range)
**Cohort:** Post-Rituximab, Early stage only **Factor:** Chemo vs. CMT	0.013	**No RT:** 4 (3–6)	**CMT:** 6 (4–6)
**Cohort:** Post-Rituximab, Advanced stage only **Factor:** Chemo vs. CMT	0.972	**No RT:** 6 (no range)	**CMT:** 6 (no range)

### 3.6 Impact of treatment strategy on CR in patients with GI-DLBCL

Encompassing patients treated pre- and post-rituximab, 168 patients (82% of the cohort) experienced complete response (CR). The CR rate was 81% among evaluable patients after first-line chemotherapy, 89.8% after RT when given in CMT, and 39% after second line chemotherapy.

Of 38 patients who were treated in the pre-rituximab era, 52.6% (*n*=20) received RT. In this pre-rituximab era, 78.9% of patients (*n*=30) experienced CR after systemic therapy. Eight patients (21.1%) had PR, of whom 2 received RT—one of these patients eventually developed CR. The remaining 7 patients who had PR did not achieve CR at any timepoint. All 8 patients eventually experienced progression of disease, including the patient who had a CR following RT; they developed an out-of-field recurrence.

In the post-rituximab era, the majority (87%) had CR after systemic therapy only. Despite RT being associated with fewer systemic therapy cycles, among patients in the post-rituximab era with limited stage disease who had a CR following systemic therapy (*n*=72), we saw that OS (*p*=0.41) and PFS (*p*=0.71) did not differ with and without consolidative RT, **Table 3**. Four patients with early stage disease had PET-based PR after completion of chemotherapy with Deauville Scores (DS) of 4 (R-CHOP x3 cycles in 1 patient, and x6 cycles in 3 patients) and were treated with ISRT. Two patients with PR after chemotherapy (50%) experienced subsequent CR after RT (DS of 1-3) to 30.6 or 36 Gy in 17 or 20 fractions, respectively. The other two patients post-RT had PR (*n*=1, 30.6 Gy in 17 fractions) or NR (*n*=1, 30 Gy in 10 fractions). In the event of PR after initial systemic therapy, 3 patients who were not dispositioned to RT instead underwent CAR-T (*n*=1), 2 cycles of RICE then 2 R-DHAP (*n*=1) with continued progression of disease to which they succumbed; or were lost to follow-up (*n*=1). The patient managed with CAR-T therapy subsequently maintained a stable disease status.

**Table 3:** Univariate analysis of patient, disease, and treatment factors associated with decrements or improvements in overall and progression-free survival.

Factor	Overall Survival *p*-value (HR, 95% CI)	Notes	Progression-Free Survival *p*-value (HR, 95% CI)	Notes
**Sex**	0.31		0.06	
**Genetics**	0.055	DH has ⇣ OS compared to DE [*p*=0.014, 10.06 (1.05-96.76)]; median OS 57 mo with DH	0.029	DH has ⇣ PFS than DE. *p*=0.03 [4.45 (1.11-17.88)]
**CR with therapy?**	<0.001 0.08 (0.04-0.17)	⇣ if never achieved CR; median OS = 23 mo if no CR	<0.001 0.14 (0.08-0.26)	⇣ if never achieved CR
**GCB status**	0.46		0.72	
**No. of systemic lines**	<0.001 2.92 (1.97-4.32)	⇣ with more systemic lines	<0.001 4.14 (2.97-5.76)	⇣ PFS with more systemic lines
**IPI**	0.001	⇣ with increasing IPI IPI 1: *p=*0.010.07 (0.01-0.55)	<0.001	⇣ with increasing IPI IPI 1: *p=*0.004 0.25 (0.09-0.64)
**Chemo alone vs. CMT**	0.35		0.28	
**ECOG**	0.31		0.38	
**Stage**	0.002	Stage I with ↑ OS than all others II: *p*=0.006 3.85 (1.17-12.64) III: *p=*0.001 7.09 (1.36-37.09) IV: *p*<0.001 5.23 (1.88-14.58)	<0.001	Stage I with ↑ PFS than all other stages II: *p*=0.018 2.56 (1.01-6.52) III: *p=*0.001 5.81 (1.58-21.35) IV: *p*<0.001 4.63 (2.18-9.82)
**Site** (stomach vs. all)	0.32		0.31	
**Age at dx**	0.82		0.82	
**SUV_max_ at dx**	0.31		0.41	
**Disease size (cm)**	0.14		0.26	
**Bulky vs. non-bulky**	0.09		0.038 2.52 (1.15-5.52)	⇣ PFS with bulky disease
**Pre-Rituximab vs. Post**	0.83		0.89	
COHORT STUDIES				
**Cohort:** all CMT only **Factor:** Site (stomach vs. all)	0.04 4.21 (0.94-18.86)	Stomach as treated site of disease associated with ↑ OS	0.037 3.34 (0.99-11.20)	Stomach as treated site of disease associated with ↑ PFS
**Cohort:** Early stage only **Factor:** Chemo vs. CMT	0.94		0.46	
**Cohort:** Advanced stage only **Factor:** Chemo vs. CMT	0.96		0.076	
**Cohort:** Bulky **Factor:** Chemo vs. CMT	0.98		0.78	
**Cohort:** Pre-Rituximab **Factor:** Chemo vs. CMT	0.22		0.009 0.06 (0.01-0.49)	CMT associated with ↑ PFS (270 mo vs. 102 mo)
**Cohort:** Post-Rituximab **Factor:** Chemo vs. CMT	0.44		0.86	
**Cohort:** Post-R, Early stage **Factor:** Chemo vs. CMT	0.98		0.74	
**Cohort:** Post-R, Advanced stage **Factor:** Chemo vs. CMT	0.08		0.008 3.59 (1.32-9.75)	CMT associated with ⇣ PFS (19 vs. 96 mo)
**Cohort:** No CR **Factor:** Chemo vs. CMT	0.09		0.32	
**Cohort:** Post-R, No CR, Early stage **Factor:** Chemo vs. CMT	0.797		0.212	
**Cohort:** Post-R, CR, Early stage **Factor:** Chemo vs. CMT	0.41		0.710	
**Cohort:** Pre-R, Early stage **Factor:** Chemo vs. CMT	0.19		0.002 0.06 (0.01-0.47)	CMT associated with ↑ PFS
**Cohort:** Pre-R, Advanced stage **Factor:** Chemo vs. CMT	0.48		0.81	
**Cohort:** Bulky, Early stage **Factor:** Chemo vs. CMT	0.63		0.41	
**Cohort:** Bulky, Advanced stage **Factor:** Chemo vs. CMT	0.52		0.41	
**Cohort:** Non-bulky, Early stage **Factor:** Chemo vs. CMT	0.72		0.99	
**Cohort:** Non-bulky, Advanced **Factor:** Chemo vs. CMT	0.87		0.25	
**Cohort:** Bulky, Post-R **Factor:** Chemo vs. CMT	0.67		0.49	

↑, increased/improved; ↓, decreased/worse.

### 3.7 Comparison of clinical outcomes by disease stage for patients with GI-DLBCL

Patients with early-stage disease had longer OS and PFS (*p*<0.005 for both) with median survival not reached in early-stage disease and 175 months with advanced stage. When stratified by disease stage, 2-year OS (**Figure 2B**) and PFS (**Figure 2E**) were 93% (87–97) and 92% (85–95) for patients with early-stage disease, vs. 2-year OS at 85% (74–92) and PFS at 77% (65–85) for advanced stage disease (*p*<0.005 for both). Five-year OS and PFS were 90% (83–95) and 86% (78–91) for early-stage disease, and 67% (50–79) and 57% (43–69) for advanced-stage disease. Ten-year OS and PFS were 86% (73–93) and 73% (55–85) for early-stage disease, but could not be computed for advanced stage disease. For the post-rituximab era only cohort, findings were consistent with early-stage disease (*n*=88) associated with longer OS (**Supplementary Figure 3B**) and PFS (**Supplementary Figure 3E**) compared to in patients with advanced stage disease (*n*=62), *p*<0.005 for both. Two- and 5-year OS were both 94% (86-97) for patients with early-stage disease, and 81% (67-89) and 64% (44-78) for patients with advanced-stage disease. Two- and 5-year PFS were 91% (82-96) and 88% (76-94) for patients with early-stage disease, and 60% (45-72) and 56% (40-69) for patients with advanced-stage disease. There were too few events of advanced stage disease in the CMT cohort (*n*=2) to render informative analysis of survival stratified by stage.

### 3.8 Characterization of outcomes in patients with GI-DLBCL with disease bulk

Fifty patients presented with bulky disease (≥7.5 cm), of whom 18 (36%) had early stage disease. Among patients with bulky disease, 5 (10%) developed relapsed disease at the initial site of disease bulk. Four of the 5 patients did not receive consolidative radiation. Among these 4 patients, 3 of the 4 relapsed only in their initial site of bulky disease in the stomach (*n*=2) or stomach and mesentery (*n*=1).

Among patients with early stage bulky disease, 5 underwent consolidative RT (27.8%), all of whom were treated to the initial site of bulky disease. Two of these patients received abbreviated courses of R-CHOP (3 and 4 cycles) followed by ISRT to initially bulky disease in the stomach. Two received 6 cycles of R-CHOP with a metabolic PR, and were consolidated with ISRT to the stomach or pancreas, respectively. The remaining patient received 6 cycles of R-CHOP with a CR followed by ISRT to the stomach. None of these 5 patients experienced disease relapse after treatment. Among patients with bulky advanced stage disease (*n*=31, 62%), only 4 received RT, with 2 of these 4 patients receiving RT to sites of bulky disease. Two of 19 patients with initially bulky disease in the stomach not treated with consolidative RT subsequently experienced disease relapse in the stomach only.

### 3.9 Univariate and multivariable analysis of factors associated with worse OS and PFS in GI-DLBCL

Factors associated with worse OS (**Table 3**, second column) include double-hit lymphoma (*p=*0.014), higher IPI (*p*=0.001), and incomplete response to therapy (*p*<0.001, median OS of 23 months). Patients with early stage disease had better prognosis compared to those with advanced stage disease (advanced stage hazard ratio (HR) of 3.2 (95% CI: 1.5–6.9, *p*=0.005) with respect to OS, and 3.7 (1.9–7, *p*<0.0001) with PFS). Gender, age, disease bulk, immunophenotype, GI site, SUV_max_, or receipt of RT were not significantly associated with survival outcome.

Factors uniquely correlated with inferior PFS (**Table 3**, fourth column) included disease bulk (*p*=0.038), for which OS only approached significance (*p*=0.09). Factors associated with worse PFS were otherwise identical with those associated with worse OS, and included high grade B cell lymphoma with *Myc/Bcl2* translocations (*p*<0.03), higher IPI (*p*<0.001), more lines of therapy received (*p*<0.001), and not experiencing CR (*p*<0.001, median OS of 15 months). Stage I disease was associated with improved PFS compared to all other stages, *p*<0.012. For OS, HR for treatment with chemotherapy alone vs. CMT for OS and PFS were 1.5 (95% CI: 0.7–3.1, *p*=0.35), and 1.45 (0.78–2.7, *p*=0.26), respectively.

Among patients treated with CMT, 77% had radiation directed to the stomach. OS and PFS were longer in patients who received RT directed to the stomach vs. all other sites, *p ≤* 0.04 for both (**Table 3**, bottom panel). Of note, more generally, disease involving the stomach vs. other sites was not associated with improved OS or PFS, **Table 3**.

In the pre-rituximab era, CMT was associated with improved PFS (270 vs. 102 months), *p*=0.009 (**Table 3**) but not OS. Improved PFS with CMT was significant for patients with limited stage disease (*n*=29) (*p*=0.002), but not significant (*p*=0.81) in patients with advanced stage disease (*n*=6). Patients with advanced stage disease treated with chemotherapy and radiation had worse survival outcomes (**Table 3**), however, all 6 patients with advanced stage disease received salvage/palliative RT for gross disease and half were treated with palliative intent. Specifically in the post-rituximab cohort, 6 patients (10%) with advanced disease underwent chemoradiation, whereas 54 patients received systemic therapy alone. Of these 6 patients, 4 (67%) received palliative RT to extraluminal metastatic sites.

On multivariable analysis (**Table 4**), worse OS was associated with not having CR (HR: 9.56, 95% CI: 4.3–21.3, *p*<0.001) and with stage IV disease at diagnosis (HR: 3.8, 95% CI: 1.35–10.8, *p*=0.01). Only advanced stage disease was associated with worse PFS on multivariable analysis (**Table 4**). On repeating the analysis to only include patients in the post-rituximab era, both worse OS and PFS were associated with not having CR (HR: 0.1, 95% CI: 0.05–0.38 [OS] and HR: 0.2, 95% CI: 0.1–0.47 [PFS], *p*<0.001 for both) and with advanced stage disease at diagnosis (*p*<0.02 for both OS and PFS), **Supplementary Table 4**. When analyzing only patients who received upfront RT as part of CMT, no variables associated with OS decrements, and worse PFS was only associated with not achieving CR (HR: 0.02, 95% CI: 0.002–0.25, *p*<0.001), **Supplementary Table 5**.

**Table 4:** Multivariate analysis of patient, disease, and treatment factors associated with decrements in overall and progression-free survival.

Characteristic	OS HR	95% CI	*p*-value	PFS HR	95% CI	*p*-value
Stage						
I	*Ref*			*Ref*		
II	2.08	0.63 – 6.91	0.23	2.9	1.2 – 7.1	**0.023**
III	4.6	0.85 – 24.8	0.076	5.9	1.6 – 21.8	**0.007**
IV	3.8	1.35 – 10.8	0.012	4.7	2.2 – 9.9	**<0.001**
Achieved CR?						
Yes	9.6	4.3 – 21.3	**<0.001**	—	—	

### 3.10 Prevalence and type of acute and late toxicities

Eighty-four (41.2%) patients had acute Grade 3 toxicities, and 12 (5.9%) patients had late Grade 3 toxicities (**Table 5**). Treatment with chemotherapy alone and IPI of 2–5 were more likely to be associated with Grade 3 acute toxicities (*p*<0.03). ECOG of 0–1 was associated with a lower likelihood of acute and late toxicities (*p ≤* 0.005).

**Table 5:** Univariate analysis of patient factors associated with acute and late toxicities.

Acuity	Factor	*p*-value	Category	*n* (%) among G3 toxicities in category
**Acute** *n*=84 (41.2%) patients with G3 toxicities	Early vs. advanced	0.094		
	Chemo vs. CMT	0.025	Chemo	65 (77.4%)
			CRT	19 (22.6%)
	No. of lines	0.13		
	IPI	0.026	IPI 0	8 (9.5%)
			IPI 1	23 (27.4%)
			IPI 2	24 (28.6%)
			IPI 3	17 (20.2%)
			IPI 4	9 (10.7%)
			IPI 5	4 (4.8%)
	Gender	0.075		
	ECOG	0.005	ECOG 0	16 (19.0%)
			ECOG 1	53 (63.1%)
			ECOG 2	10 (11.9%)
			ECOG 3	2 (2.4%)
			ECOG 4	3 (3.6%)
	Stage	0.227		
	Age	0.169		
	SUV_max_	0.342		
**Late** *n*=12 (5.9%) patients with G3 toxicities	Early vs. advanced	0.363		
	Chemo vs. CMT	0.068		
	No. of lines	0.615		
	IPI	0.055		
	Gender	0.126		
	ECOG	<0.001	ECOG 0	1 (8.3%)
			ECOG 1	6 (50.0%)
			ECOG 2	2 (16.7%)
			ECOG 3	1 (8.3%)
			ECOG 4	2 (16.7%)
	Stage	0.788		
	Age	0.756		
	SUV_max_	0.418		

**Figure 4:**
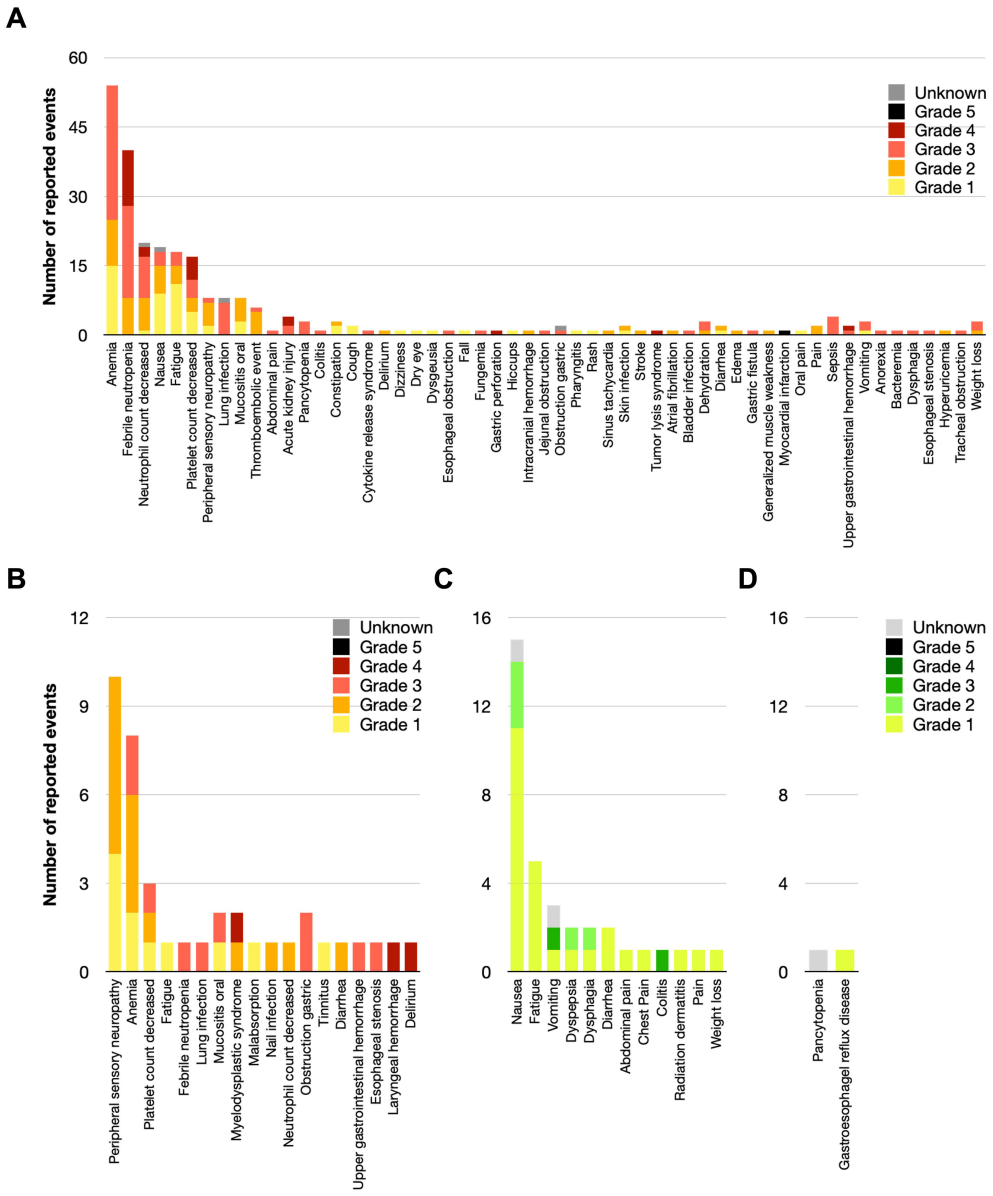
Provider-reported treatment-related toxicities in the treatment of DLBCL of the GI tract. Acute **(A)** and chronic **(B)** chemotherapy-associated toxicities graded by the CTCAE v5.0, as well as acute **(C)** and chronic **(D)** radiation-related toxicities are shown below.

Prevalent acute treatment-related toxicities associated with systemic therapy (**Figure 4A**) included anemia (26.3% of the patients, of which 54% were G3, 19% G2, and 27% G1), febrile neutropenia (19.5% of the patients, of which 30% G4, 50% G3, and 20% G2), nausea (9% of the patients, of which 16% G3, 32% G2, 47% G3, 5% unknown), fatigue (8.8% of the patients, of which 17% G3, 22% G2, 61% G1), and thrombocytopenia (8.3% of the patients, of which 29.5% G4, 23.5% G3, 17.6% G2, 29.5% G1). The most common late toxicities from systemic therapy included peripheral neuropathy (4.9% of the patients, of which 60% Grade 2, 40% Grade 1) and anemia (4% of the patients, of which 25% Grade 3, 50% Grade 2, 25% Grade 1), **Figure 4B**.

RT-associated acute toxicities were less common, but specifically included nausea (6.8% of the patients, of which 20% G2, 73% G1, 7% unknown) and fatigue (2.4% G1), **Figure 4C**. CMT was safe for patients, with late toxicities attributed to RT being exceedingly rare (**Figure 4D**).

A list of late or chronic treatment-related toxicities is detailed in **Supplementary Table 6**, organized by disease location in the stomach, small bowel, or large bowel. Toxicities identified included rare events of perforation, obstruction, GI bleeding, fistula, pyloric stenosis, malabsorption, chronic anemia, or requirement of surgical intervention.

## 4 Discussion

Although primary GI tract lymphomas are rare malignancies, their incidence is rising (8–10). Among extranodal sites involved by NHLs, the GI tract is the most frequent site (11). Herein, we demonstrate in a large cohort of 204 patients, that contemporary treatment of GI-DLBCL results in favorable outcomes and minimal late toxicity. OS and PFS is excellent in treated GI-DLBCL irrespective of treatment with chemotherapy alone or CMT. In patients with early stage disease, treatment with RT can successfully be offered with an abridged systemic regimen in appropriately selected patients. We found equivalent outcomes with fewer cycles of systemic therapy and RT consolidation vs. longer regimens of systemic therapy—RT was well-tolerated and may allow for potential sparing of toxicity that may come from additional chemotherapy cycles.

The role for consolidative RT in DLBCL has evolved over time, but has been well-established to improve event-free survival for bulky disease or bony site involvement (4–6, 12), palliative/bridging prior to CAR-T cell therapy (13–15), or for limited-stage and non-bulky disease if patients are treated with an abbreviated course of ST with only 3 cycles of R-CHOP (16). Regarding the latter, the SWOG 8736 trial demonstrated that for patients with Stage I-IE and non-bulky Stage II-IIE DLBCL, an abbreviated course of chemotherapy (CHOP ×3) followed by involved-field RT provided comparable long-term outcomes to a more extended course of chemotherapy (CHOP ×8) (16). The FLYER trial (17) did not investigate RT in either of its arms, but established the noninferiority of R-CHOP ×4—R ×2 vs. R-CHOP ×6 in patients with limited-stage, non-bulky, low-risk DLBCL, and reinforced a platform for omission of RT compared to historical R-CHOP ×3 + RT. These studies collectively highlight that systemic therapy plays a central role in DLBCL treatment, with RT being considered on a case-by-case basis for specific indications or delivered with an abridged chemotherapy regimen. In our study, patients with early-stage DLBCL were mostly like to receive CMT as part of abbreviated systemic therapy courses.

While CMT provided a PFS benefit in the pre-rituximab era in this cohort, this was not apparent in the overall cohort treated post-rituximab. This is consistent with most patients in this study treated with CMT in the early stage consolidative setting. While CMT was associated with worse PFS among patients with advanced stage disease in the post-rituximab era compared with chemotherapy alone, this was likely due to the predominantly palliative use of RT among advanced stage patient in this study.

With regards to clinical factors such as bulky disease or incomplete response to chemotherapy, although the numbers in our study were small making statistical comparison challenging, it appears RT may improve the associated risks of relapse. Among patients with bulky disease, approximately 10% relapsed in the initial site of disease bulk. Four of 5 patients who relapsed did not receive consolidative RT, and the pattern of relapse in 3 of 4 patients was localized to the initial site of disease bulk, suggesting potential benefit for local therapy. Similarly, although only few patients were consolidated with RT (typically ≥30 Gy) after partial response to chemotherapy, this was a strategy that afforded several patients excellent treatment outcome.

Concerns exist regarding the possible complications of RT when directed toward the GI tract, such as perforation and bleeding. However, we found RT to be well-tolerated. Of the 4 patients with perforation in our series (2.1% of luminal cases), only 1 received RT. In a prior study, severe complications necessitating emergency surgery were reported in less than 5% of patients (18). In our series, there were expected acute adverse effects with systemic therapy and a low rate of late toxicities. Toxicities attributed to RT were even rarer. Supporting this finding, typical doses used for the treatment of DLBCL are well below the standard dose constraints commonly cited for the luminal bowel.

In considering the value of this work relative to the published body of literature, strengths of our study include the large size of the cohort and extended follow-up period. Limitations include this being a retrospective study subject to selection biases that may have affected patient disposition, introduced irregularities in follow-up, or compounded heterogeneity. We acknowledge that uniformity in our cohort analyses could have been improved by adjusting for age or stage across all of the cohorts; stage was a controlled variable for several but not all of the analyses.

In summary, we demonstrate that patients with DLBCL of the GI system have favorable outcomes, with well tolerated treatment and minimal late toxicity. There were no survival differences with CMT compared to chemotherapy alone, but CMT may reduce the need for additional chemotherapy cycles for selected early stage patients and consequently mitigate adverse effects in these patients. Furthermore, CMT can be considered to mitigate the risks of bulky and incompletely responding disease.

## Data Availability

The raw data supporting the conclusions of this article will be made available by the authors, without undue reservation.
