# Supplementary material for: Outcomes and toxicities in patients with diffuse-large B cell lymphoma involving the gastrointestinal tract and digestive organs
Source: Front Oncol. 2024 Sep 11;14:1447020. doi: 10.3389/fonc.2024.1447020 (PMC11422352; doi:10.3389/fonc.2024.1447020)
Supplement: Supplementary file 1 [file DataSheet1.pdf]

Supplementary Material

Supplementary Figure 1. Patient inclusion and exclusion criteria. 85 patients were excluded owing to a number of factors, yielding a final cohort of 204 patients who were analyzed. *Abbreviations:* RT = radiation therapy.

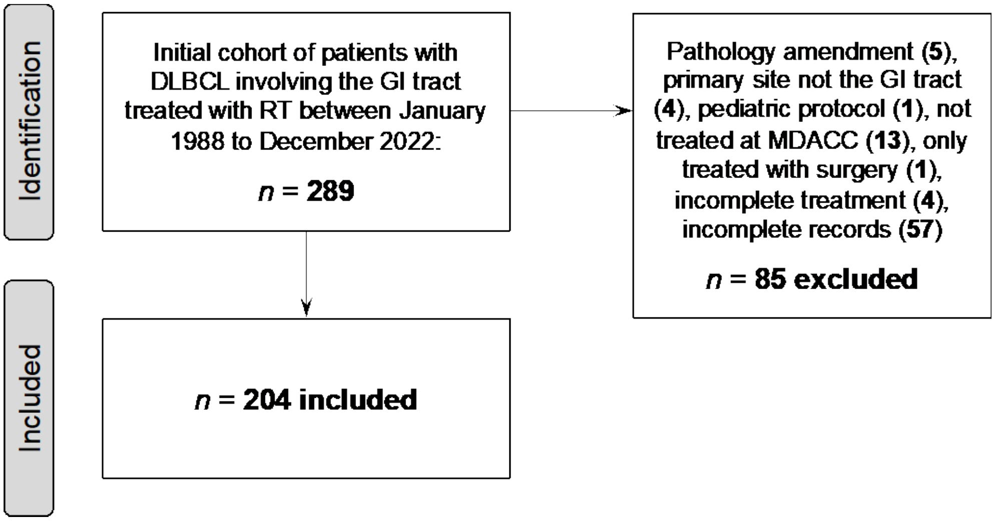

Supplementary Figure 2. Gastrointestinal comorbidities among all patients. The most common comorbidities included GERD, followed by colorectal cancer, and liver disease.

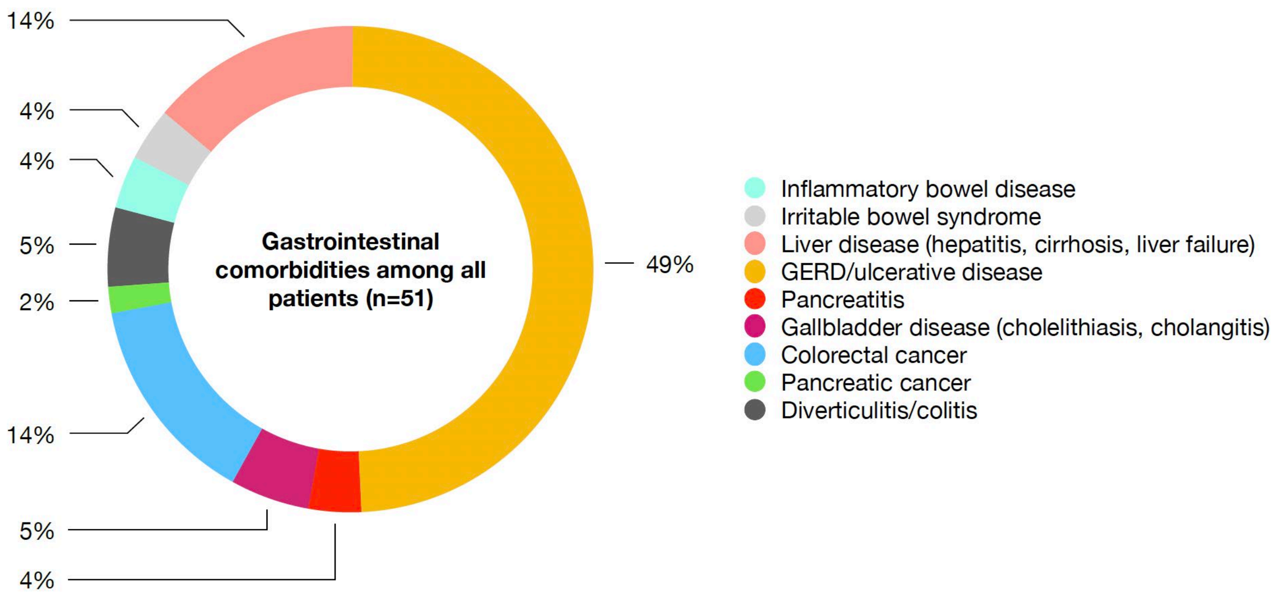

Supplementary Figure 3. Survival outcomes of patients with DLBCL of the GI tract treated only in the post-rituximab era. Overall survival of the full post-rituximab cohort **(A)**, stratified by stage **(B)**, and receipt of chemotherapy vs. chemoradiation **(C)**. Also depicted are progression-free survival of the full post-rituximab cohort **(D)**, as well as analyses stratified by stage **(E)**, and chemotherapy vs. chemoradiation disposition **(F)**. *p<0.05, **p<0.01, ***p<0.001, ****p<0.0001.

POST-RITUXIMAB COHORT ONLY

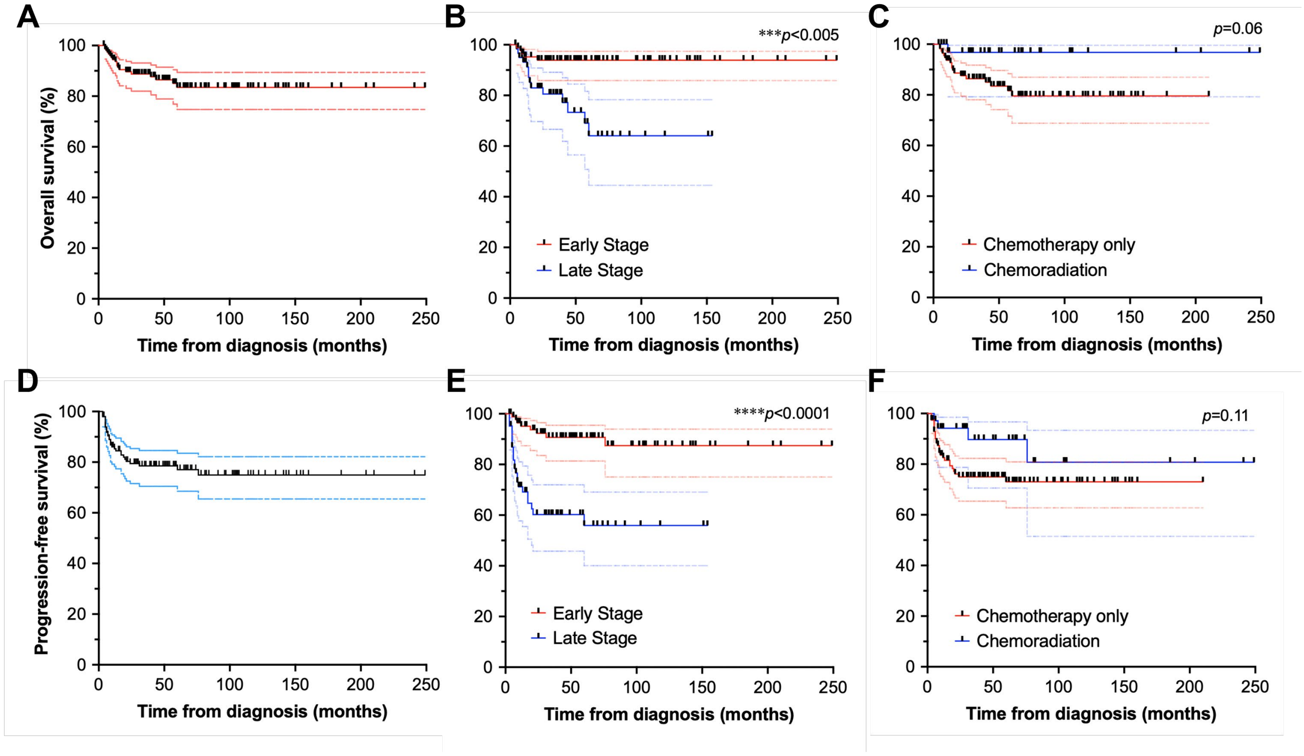

Supplementary Figure 4. Survival outcomes of patients with DLBCL of the GI tract treated with CMT. Patients treated with systemic therapy alone were excluded from this analysis. Overall survival of the full CMT cohort **(A)**, also stratified by stage **(B)**. Also depicted are progression-free survival of the full CMT cohort **(C)**, as well as an analysis stratified by stage **(D)**. **p*<0.05, ***p*<0.01, ****p*<0.001, *****p*<0.0001.

COMBINED MODALITY THERAPY (CMT) COHORT ONLY

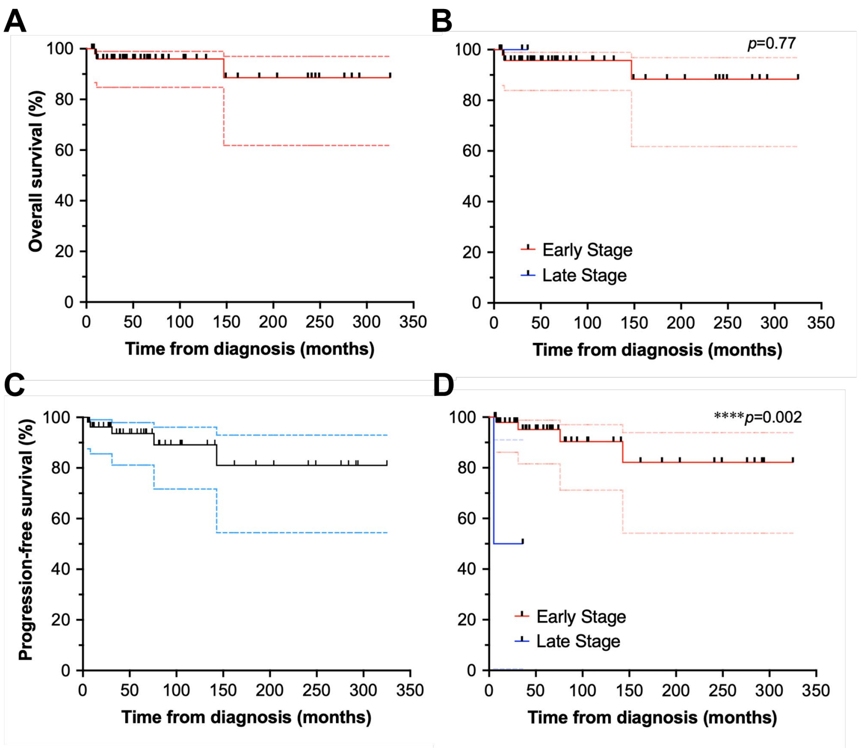

Supplementary Table 1. Summary of chemotherapies received.

	**All patients,** *n*=204 *n* (%)	**Early stage DLBCL,** *n*=125 (61.3%) *n* (%)	**Advanced stage DLBCL,** *n*=75 (36.8%) *n* (%)
**Systemic line 1**			
Without rituximab	Subtotal: 38 (18.6)	Subtotal: 31 (24.8)	Subtotal: 8 (10.7)
**CHOP**	31 (15.2)	27 (21.6)	4 (5.3)
**EPOCH**	3 (1.5)	1 (0.8)	2 (2.7)
**HCVAD**	4 (2.0)	3 (2.4)	2 (2.7)
With rituximab	Subtotal: 151 (74.0)	Subtotal: 87 (69.6)	Subtotal: 61 (81.3)
**R-CHOP**	114 (55.9)	75 (60.0)	37 (49.3)
**R-EPOCH**	31 (15.2)	9 (7.2)	22 (29.3)
**R-HCVAD**	6 (2.9)	3 (2.4)	2 (2.7)
Other	15 (7.4)	5 (4.0)	6 (8.0)
**Systemic line 2**			
Without rituximab	Subtotal: 2 (1.0)	Subtotal: 1 (0.8)	Subtotal: 1 (1.3)
**CHOP**	2 (1.0)	1 (0.8)	1 (1.3)
**EPOCH**	0 (0.0)	0 (0.0)	0 (0.0)
**HCVAD**	0 (0.0)	0 (0.0)	0 (0.0)
With rituximab	Subtotal: 8 (3.9)	Subtotal: 3 (2.4)	Subtotal: 5 (6.7)
**R-CHOP**	3 (1.5)	1 (0.8)	2 (2.7)
**R-EPOCH**	5 (2.5)	2 (1.6)	3 (4.0)
**R-HCVAD**	0 (0.0)	0 (0.0)	0 (0.0)
Other	37 (18.1)	13 (10.4)	24 (32.0)

Supplementary Table 2. Presenting symptoms at diagnosis by GI-DLBCL subsite.

**GI-DLBCL LOCATION**		**Stomach** (*n*=124)	**Small Intestine** (*n*=44)	**Large Intestine** (*n*=23)
**INITIAL PRESENTATION** *n* (%)	Obstruction***	5 (4.1%)	11 (25%)	—
	GI bleeding	19 (15.4%)	5 (11.4%)	4 (17.4%)
	Abdominal pain	45 (36.6%)	13 (29.5%)	8 (34.8%)
	GERD	3 (2.4%)	—	2 (8.7%)
	Perforation	—	2 (4.5%)	—
	Nausea/vomiting	2 (1.6%)	1 (2.3%)	—
	Chronic anemia	1 (0.8%)	—	—
	Weight loss	—	—	1 (4.3%)
	Misc.	—	—	1 (4.3%)[†]

***$p<0.001$, [†]Ulcerative colitis requiring elective colectomy upon which GI-DLBCL was identified

Supplementary Table 3. Radiation treatment details.

		**All patients,** *n*=65 *n* (%)	**Early stage DLBCL,** *n*=54 (83.1%) *n* (%)	**Advanced stage DLBCL,** *n*=9 (13.8%) *n* (%)
**Radiation intent**	**Curative**	55 (84.6)	50 (92.6)	4 (44.4)
	**Palliative**	8 (12.3)	4 (7.4)	4 (44.4)
	**Missing**	2 (3.1)	0 (0.0)	1 (11.1)
**Radiation context**	**Initial therapy (upfront)**	57 (87.7)	52 (96.3)	4 (44.4)
	**Disease recurrence**	5 (7.7)	1 (1.9)	4 (44.4)
	**Missing**	3 (4.6)	1 (1.9)	1 (11.1)
	**Consolidation**	51 (78.5)	47 (87.0)	2 (22.2)
	**Gross disease**	14 (21.5)	7 (13.0)	6 (66.7)
	**Missing**	0 (0.0)	0 (0.0)	1 (11.1)
**Site(s) of radiation**	**Stomach**	50 (76.9)	45 (83.3)	2 (22.2)
	**Small intestines**	2 (3.1)	2 (3.7)	0 (0.0)
	**Pancreas**	1 (1.5)	1 (1.9)	0 (0.0)
	**Abdomen/pelvis, nodes**	9 (13.8)	8 (14.8)	1 (11.1)
	**Other** (thorax, scrotum, bone, CNS)	6 (9.2)	0 (0.0)	6 (66.7)
	**Missing**	1 (1.5)	0 (0.0)	1 (11.1)
**Concurrent chemotherapy**	**Yes**	0 (0.0)	0 (0.0)	0 (0.0)
	**No**	63 (96.9)	54 (100.0)	8 (88.9)
	**Missing**	2 (3.1)	0 (0.0)	1 (11.1)
**Dose of RT**	**Median** (IQR)	36 (30.6-39.6)	36 (30.6-39.6)	34 (30.5-37)
	**Missing**	7 (10.8)	5 (9.3)	1 (11.1)
**Fractionation**	**Median** (IQR)	18 (17-22)	20 (17-22)	16.5 (15-15)
	**Missing**	7 (10.8)	5 (9.3)	1 (11.1)
**Technique**	**2D/3D**	33 (50.8)	27 (50.0)	5 (55.6)
	**IMRT/VMAT**	25 (38.5)	22 (40.7)	3 (33.3)
	**Missing**	9 (13.8)	6 (11.1)	2 (22.2)

Supplementary Table 4. Multivariate analysis of patient, disease, and treatment factors associated with decrements in overall and progression-free survival among patients treated with rituximab.

Characteristic	OS HR	95% CI	*p*-value	PFS HR	95% CI	*p*-value
**Stage**						
I	*Ref*			*Ref*		
II	12.2	1.34 – 112.34	**0.027**	2.4	0.63 – 9.12	0.200
III	20.2	1.75 – 233.05	**0.016**	7.1	1.65 – 30.35	**0.008**
IV	12.9	1.62 – 102.22	**0.016**	5.8	2.15 – 15.85	**<0.001**
**Achieved CR?**						
Yes	0.1	0.05 – 0.38	**<0.001**	0.2	0.10 – 0.47	**<0.001**

Supplementary Table 5. Multivariate analysis of patient, disease, and treatment factors associated with decrements in progression-free survival among patients treated with combined modality therapy.

Characteristic	PFS† HR	95% CI	*p*-value
**Achieved CR?**			
Yes	0.02	0.002 – 0.254	**0.002**

†No variables associated with OS decrements among the CMT cohort (*n*=55), hence only PFS is shown

Supplementary Table 6. Late treatment-related complications by GI-DLBCL subsite.

GI-DLBCL LOCATION		Stomach (*n*=124)	Small Intestine (*n*=44)	Large Intestine (*n*=23)
TREATMENT COMPLICATION *n* (%)	Perforation	3 (2.4%)	1 (2.3%)	—
	Obstruction	8 (6.5%)	5 (11.4%)	2 (8.7%)
	Bleeding	4 (3.2%)	4 (9.1%)	1 (4.3%)
	Pyloric Stenosis	1 (0.8%)	—	—
	Malabsorption	1 (0.8%)	—	—
	Chronic Anemia	1 (0.8%)	4 (9.1%)	2 (8.7%)
	Dysphagia	1 (0.8%)	—	—
	Fistula	—	1 (2.3%)	1 (2.3%)
	Surgical Intervention	***n*=9**; Dilation (*n*=1), Partial gastrectomy (*n*=2), Jejuno— or Gastrostomy (*n*=2), Unknown (*n*=4)	***n*=4**; Venting Tube/J—tube/gastrojejunostomy (*n*=1), Unknown (*n*=3)	Not specified (***n*=2**)
